# *DNMT3A* low-expression is correlated to poor prognosis in childhood B-ALL and confers resistance to daunorubicin on leukemic cells

**DOI:** 10.1186/s12885-023-10724-6

**Published:** 2023-03-18

**Authors:** Weijing Li, Shugang Liu, Chanjuan Wang, Lei Cui, Xiaoxi Zhao, Wei Liu, Ruidong Zhang, Zhigang Li

**Affiliations:** 1 Laboratory of Hematologic Diseases, Beijing Pediatric Research Institute, Beijing Children’s Hospital, Capital Medical University, National Center for Children’s Health, Beijing, China; 2 Beijing Key Laboratory of Pediatric Hematology-Oncology, Beijing, China; 3 National Key Discipline of Pediatrics, Capital Medical University, Beijing, China; 4 Key Laboratory of Major Diseases in Children, Ministry of Education, Beijing, China; 5 Hematology Oncology Center, Beijing Children’s Hospital, Capital Medical University, National Center for Children’s Health, Beijing, China; 6 Department of Hematology Oncology, Children’s Hospital Affiliated to Zhengzhou University, Zhengzhou, China

**Keywords:** Childhood B-ALL, DNMT3A expression, Genome editing of DNMT3A, DNR drug resistance

## Abstract

**Background:**

Little is known about *DNMT3A* expression and its prognostic significance in childhood B cell acute lymphoblastic leukemia (B-ALL).

**Methods:**

We determined *DNMT3A* mRNA expression in 102 children with B-ALL. Correlations with relapse-free survival (RFS) and common clinical characteristics were analyzed. *DNMT3A* was stably knocked out by CRISPR/Cas9 gene editing technology in Reh and 697 B-ALL cell lines. Cell proliferation activity after treated with daunorubicin (DNR) was determined by CCK8 assay in *DNMT3A* KO Reh and 697 cell lines.

**Results:**

*DNMT3A* expression in B-ALL patients who were in continuous complete remission (CCR) was higher than in those who got relapse (*P* = 0.0111). Receiver operating characteristic curve showed prognostic significance of *DNMT3A* expression (*P* = 0.003). Low expression of *DNMT3A* (≤ 0.197) was significantly correlated with poor RFS (*P* < 0.001) in children with B-ALL. Knock-out of *DNMT3A* in Reh and 697 cell lines significantly increased IC50 of DNR (*P* = 0.0201 and 0.0022 respectively), indicating elevated resistance to DNR.

**Conclusion:**

Low expression of *DNMT3A* associates with poor prognosis in children with B-ALL. Knock-out of *DNMT3A* confers resistance to DNR on leukemic cells.

**Supplementary Information:**

The online version contains supplementary material available at 10.1186/s12885-023-10724-6.

## Background

B cell acute lymphoblastic leukemia (B-ALL) is the most common childhood malignancy. Although cure rate of childhood B-ALL has been greatly improved with risk-adjusted therapy [1, 2], relapsed leukemia is still a leading cause of death for children mainly due to therapy resistance [2–4]. Thus, it is of great significance to clarify the mechanisms of therapy resistance and relapse of B-ALL.

DNA methyltransferase 3A (DNMT3A) catalyzes de novo DNA methylation and plays important roles in the pathogenesis of malignancies including leukemia. Furthermore, *DNMT3A* mutations in acute myeloid leukemia (AML) and T cell ALL are associated with poor prognosis of the patients [5–11]. Our previous studies have shown that *DNMT3A* mutations can be found in a few of children with B-ALL, and are correlated with poor prognosis [12]. However, the expression level of *DNMT3A* and its prognostic significance in B-ALL remains unclear.

In this study, we assessed the relationship between expression level of *DNMT3A* and prognosis in Chinese childhood B-ALL. Moreover, CRISPR/Cas9 has been used to knock out *DNMT3A* gene in leukemic Reh and 697 cell lines in order to explore the role of *DNMT3A* expression playing in resistance to chemotherapeutic drugs. We showed that low expression of *DNMT3A* was correlated with poor treatment outcome, knock-out of this gene resulted in obvious resistance to DNR, a common chemotherapeutic drug in treatment of ALL.

## Methods

### Patients

From July 2010 to May 2014, a total of 226 consecutive childhood patients with newly diagnosed B-ALL were admitted to Beijing Children’s Hospital. The criterion for the patient’s inclusion was ≥70% leukemic cells in BM samples [13, 14].

One hundred two B-ALL patients with available diagnostic bone marrow (BM) samples were enrolled in this study. One hundred twenty-four patients not fulfilling the inclusion criterion were excluded from this study. No difference was found between patients included and excluded in terms of patients’ characteristics and survival to suggest selection bias (Supplemental Table S1). BM samples from 11 patients in continuous CR were collected and used as control. ALL patients were diagnosed and treated in accordance with the Chinese Children’s Leukemia Group ALL 2008 Protocol (CCLG-ALL 2008) at Beijing Children’s Hospital [4].

Among the 102 patients, there were 64 boys and 38 girls, aged from 1 to 13 years with a median age of 4. Chromosome karyotype analysis was performed in 66 patients and the karyotype results were interpreted according to the International System for Human Gytogenomic Nomenclature guide-lines [15]. Of the 66 patients, normal and abnormal karyotype was seen in 22 and 32 patients respectively and no metaphase schizophrenia was found in the 12 patients. Fusion gene was detected in all 102 patients by a nested multiplex reverse transcription polymerase chain reaction (RT-PCR) system, as described by Gao C. et al. [16]. Thirty-four patients carried 4 types of fusion genes including *ETV6-RUNX1*, *TCF3-PBX1*, *BCR-ABL1*, and *FUS-ERG.* The details of stratification and treatment according to CCLG-ALL 2008 were described previously [4, 17]. Ninety-four patients were in continuous complete remission (CR), 8 patients relapsed 2 to 62 months after diagnosis. The follow-up time ranged from 1.67 to 92 months (median, 59 months). MRD at d33 (the end of induction of remission) and d78 (before consolidation therapy) were detected using RQ-PCR targeted at Ig/TCR (*immunoglobulin* and *T cell receptor* gene rearrangements) according to European MRD (Minimal residual disease) laboratory guidelines [18–21]. Informed consents were obtained from all the children’s parents or legal guardians.

### Cell lines

Human B-ALL cell lines Reh and HEK293T cell were purchased from National Infrastructure of Cell Line Resource (No. 3101HUMTCHu131 and 1101HUM-PUMC000010, respectively); 697 cell line was a kind gift from Dr. Suning Chen at the first affiliated Hospital of Soochow University (Suzhou, China). Reh and 697 were cultured in RPMI 1640 (GIBCO, USA) supplemented with 10% fetal bovine serum (FBS, AusGeneX, Brisbane) and 1% penicillin/streptomycin. HEK293T cell was cultured in DMEM supplemented with 10% FBS and 1% penicillin/streptomycin. All cells were maintained at 37 °C in a humidified atmosphere containing 5% CO_2_.

### Nucleic acid extraction

Mononucleated cells were separated from 1 ml of patients’ BM aspirate by centrifugation with Ficoll 400 (MD Pacific Technology CO., Ltd.) and stored at -70˚C until use. Total RNA of samples was extracted and reverse transcribed using Trizol Reagent (Invitrogen, USA) and MMLV reverse transcriptase (Promega, USA) according to the manufacturers’ instructions respectively. Genomic DNA of Reh and 697 cell lines were extracted using a Blood & Cell Culture DNA Midi Kit (TIANGEN, China) according to the manufacturer’s protocol.

### Quantitative analysis of *DNMT3A* expression

Real-time quantitative polymerase chain reaction (RQ-PCR) was performed using Power SYBR™ Green PCR Master Mix (Applied Biosystems 4,367,659) by an ABI Prism 7500 Sequence Detection System (Applied Biosystems, Foster City, CA, USA). *GUS* (β-Glucuronidase) expression was used as an internal control. The cycling condition included pre-denaturation at 95 °C for 30 s, followed by 40 cycles of 5 s at 95 °C, 30 s at 55 °C and 30 s at 72 °C. Primers were shown in Table 1. We used the cDNA samples obtained from 697 cell line as a calibrator. The relative expression of *DNMT3A* was calculated by the method of 2^−ΔΔCt^. The levels of *DNMT3A* and *GUS* were tested in triplicates.

**Table 1:** Oligo sequences

Oligo name	Sequence	Description
DNMT3A ex7 sg F	CACCGGGGGCCCGGGGAGTCTCAGA	sgRNA primer
DNMT3A ex7 sg R	AAACTCTGAGACTCCCCGGGCCCCC	
DNMT3A ex7 F	TTTCACGGCAAGGCAGCTGGTTG	PCR primer (445 bp) for T7e1 assay
DNMT3A ex7 R	AGAGGAGAGCAGGACGGGAGGAG	
DNMT3A ex23 F	GCCACCTCTTCGCTCCGCTG	RQ-PCR primer(239 bp) for clinical samples
DNMT3A ex23 R	GATGATGTCCAACCCTTTTCGCAA	
GUS F	GAAAATATGTGGTTGGAGAGCTCATT	RQ-PCR primer(101 bp) as internal control for clinical samples
GUS R	CCGAGTGAAGATCCCCTTTTTA	

The capital letters underlined indicate BsmbI sticky end

### Lenti DNMT3A-sgRNA-Cas9 constructs

**Fig. 1:**
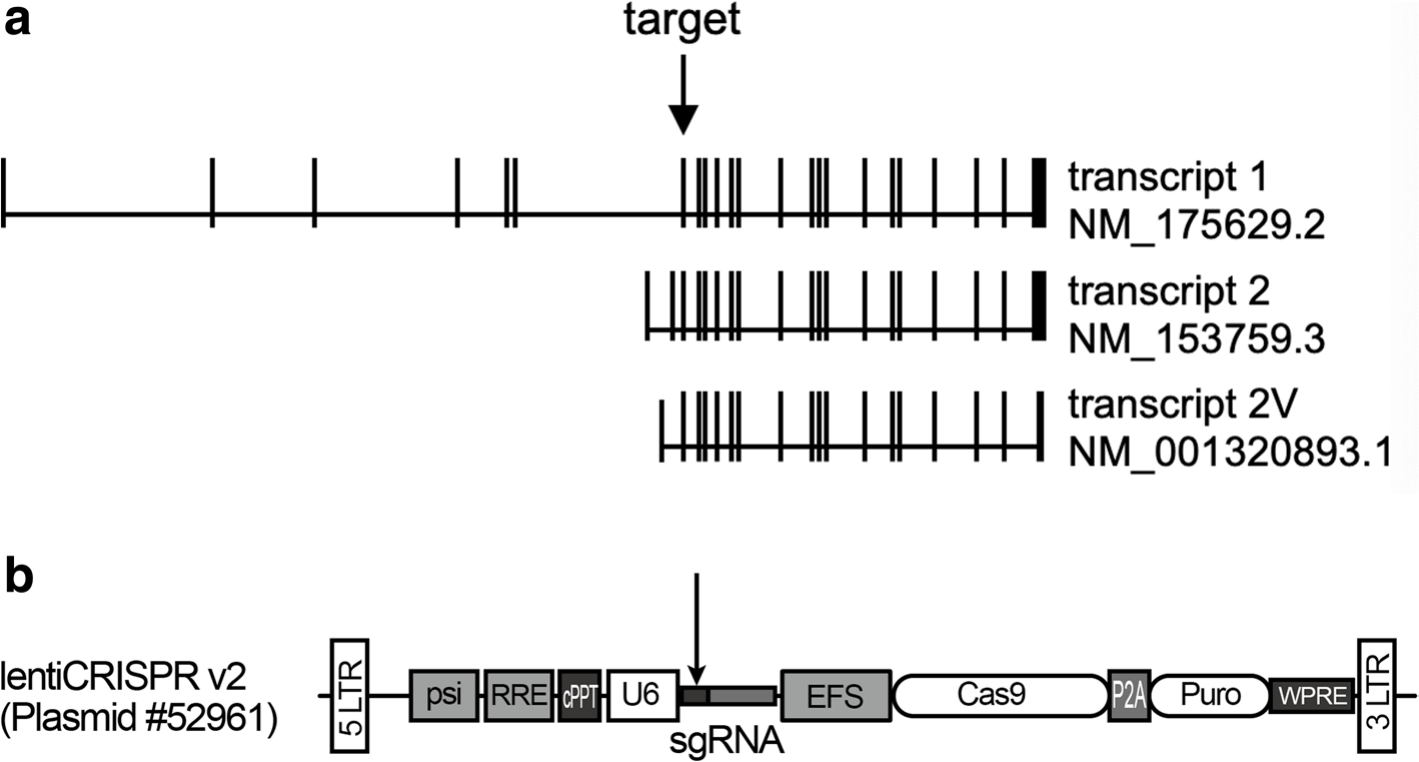
Schematic diagram of sgRNA targeting *DNMT3A*. **a** The structure of *DNMT3A* gene and the three common transcripts. Black vertical lines: exons. Horizontal lines: introns. Arrow: the location of sgRNA targeting exon 7. **b** The structure of lentiCRISPR v2 plasmid. The arrows indicate the sgRNA sequence

The cDNA sequence encoding sgRNA which targets a conserved sequence in exon 7 of human *DNMT3A* gene was synthesized and subcloned into LentiCRISPR-v2 plasmid (Addgene 52961, kindly provided by Dr. Jian Huang at Temple University, Philadelphia, PA) to make the lentiDNMT3A-sgRNA-Cas9 construct. Briefly, the forward and reverse primers including 20 bp target *DNMT3A* sequence and BsmbI sticky ends were annealed and inserted into the lentiCRISPR-v2 plasmid digested with FastDigest Esp3I (Thermo Fisher Scientific, #FD0454) (Fig. 1b). sgRNA primer sequences have been reported by Gundry MC et al. previously and were shown in Table 1 [22].

### Lentivirus production and infection

To produce lentivirus, 6 μg of transfer plasmid lentiDNMT3A-sgRNA-Cas9 or control plasmid lenti-CRISPR-v2 were co-transfected into HEK 293 T cells with 4.5 μg of packaging plasmids psPAX2 (AddGene 12260) and 3 μg of VSV-G (AddGene 8454) using FuGENE® 6 Transfection Reagent (Promega E2692) according to the manufacturer’s instructions. After incubation for 48 h, the culture supernatants containing lentivirus were harvested and filtered with 0.45 μm filter and stored at − 80 °C. The Reh and 697 cell lines (5 × 10^5^) was infected with the lentivirus at an M.O.I. of 40 separately, using spin-transduction (centrifuging the plate coated with 8 μg/ml polybrene (SANTA CRUZ) at 1200 g for 2 h at 25 °C), then were cultured for 24 h in the incubator. On the next day, the medium was changed with fresh RPMI 1640 complete medium and the cells were cultured for another 24 h.

### T7EN1 assays for quantifying frequencies of indel mutations

Lentivirus-infected cells were selected by 1 μg/ml puromycin for 2 days. Genomic DNA was extracted and used to amplify the genomic region flanking the *DNMT3A* sgRNA target site with KAPA2G Robust HotStart ReadyMix (KAPA BIOSYSTEMS KK5702) and PCR primers listed in Table 1. Then T7EN1 assay was performed using T7 Endonuclease I (NEB #M0302L) according to the Instruction Manual. The digested DNA was analyzed on electrophoresis system using a 2% agarose gel.

### Western blotting

A fraction of lentivirus-infected cells was lysed in NE-PER® Nuclear and Cytoplasmic Extraction Reagents (Thermo Fisher Scientific, USA). The lysates were denatured in 5 × SDS loading buffer by boiling at 95 °C for 10 min and were subjected on a NuPAGE™ 4–12% Bis–Tris Protein Gels (Invitrogen). After transferred to Biotrace NT nitrocellulose Transfer Membrane (PALL, 66,485), the expression of proteins was detected using following antibodies: 1:300 DNMT3A (D23G1) Rabbit mAb (No. 3598; CST) alone was incubated firstly, then 1:300 DNMT3A (D2H4B) Rabbit mAb (No. 32578; CST) and 1:2000 Lamin B1 Mouse mAb (No. 66095–1-Ig; Proteintech) were mixed and incubated together on the next day in the same blot after finishing the secondary antibody incubation and band scanning for DNMT3A(D23G1) mAb. Secondary antibody included Goat anti-Mouse IgG (H + L) Highly Cross-Adsorbed Secondary Antibody, Alexa Fluor 680 (No. A21058; Invitrogen) and Goat anti-Rabbit IgG (H + L) Highly Cross-Adsorbed Secondary Antibody, Alexa Fluor Plus 800 (No. A32735; Invitrogen). The bands were scanned by LICOR Odyssey CLX.

### Cell viability

The lentivirus-infected Reh and 697 cell lines were plated into 96-well plate separately, 10^4^/well. After treatment with 100 μl DNR, the cells were cultured for 24 h at 37 °C in a humidified atmosphere containing 5% CO_2_. The concentrations of DNR for Reh were 0.0009, 0.0055, 0.0111, 0.0222, 0.0443, 0.1773 μM; and for 697 were 0.0089, 0.0177, 0.0887, 0.1773, 0.8866, 1.7731 μM. Twenty microliters of Cell Counting Kit-8 (CCK-8, Yeasen 40203ES60*, Shanghai, China) solution were added to each well and mixed gently. After incubation for 1 h, optical density (OD) at 450 nm was determined using a Spectra MAX 190 microplate reader. After calibrated with non-cellular background, cell viability was calculated using a non-treatment control regarded as 100% of cell viability.

### Statistical analysis

Receiver Operating Characteristic (ROC) curve was used to decide the cut-off value of low- and high-expression of *DNMT3A* (*DNMT3A*^low^ and *DNMT3A*^high^) in leukemic cells of children with B-ALL. Fisher’s exact test was used to test the differences in clinical characteristics and relapse rates between *DNMT3A*^low^ and *DNMT3A*^high^ patients. Relapse free survival (RFS) was defined as the date of leukemia diagnosis to the date of recurrence. Survival estimates were calculated using the Kaplan–Meier method, and the groups were compared using the log-rank test. The independent prognostic significance of *DNMT3A* expression and the common clinical features was analyzed by Cox proportional hazards model (Method: Enter). All data were analyzed with the SPSS 16.0 software package and a *P* value < 0.05 was considered statistically significant. The fitting curves of inhibitory effects of DNR on cell proliferation were plotted by GraphPad Prism 8, and half maximal inhibitory concentration (IC50) was also calculated by the software.

## Results

### *DNMT3A* expression in childhood B-ALL

**Fig. 2:**
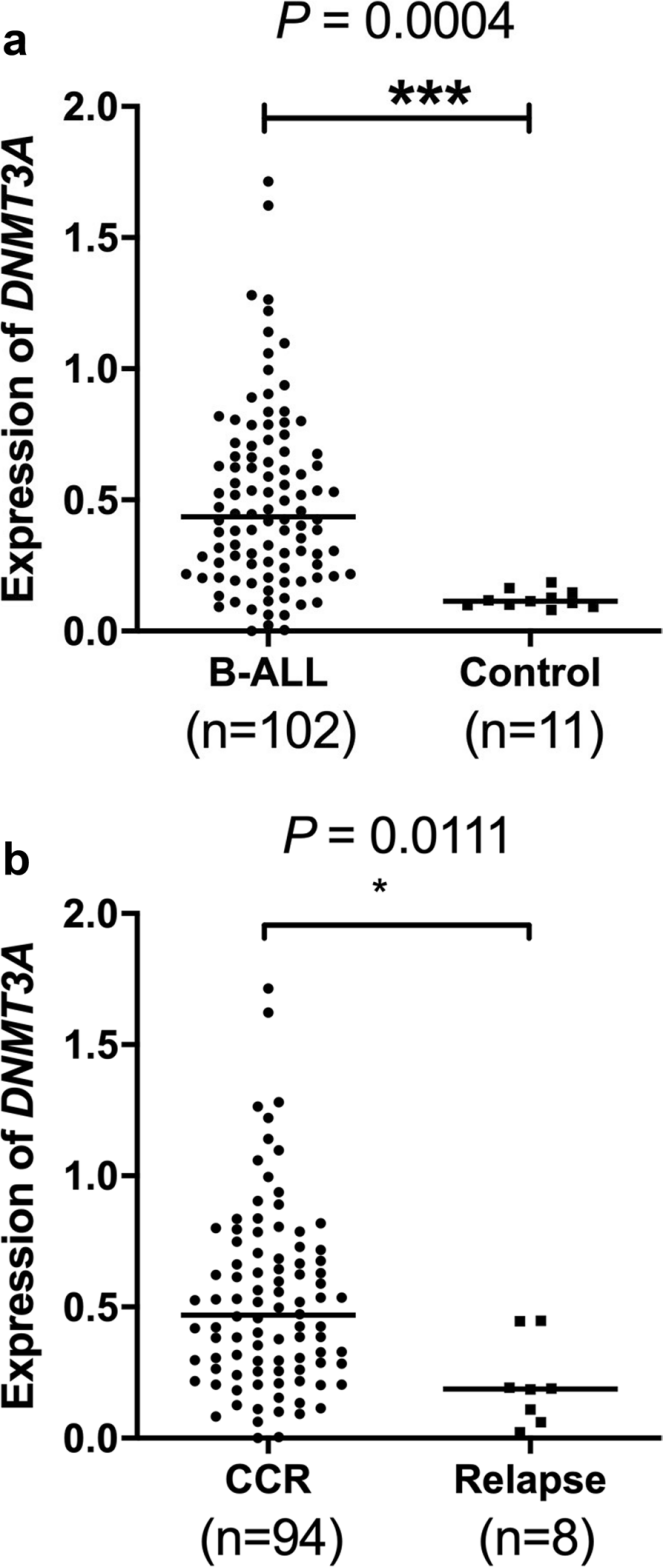
*DNMT3A* expression in ND B-ALL patients and controls. **a** *DNMT3A* expression was significantly increased in ND B-ALL patients compared with that of controls. **b** *DNMT3A* expression was significantly decreased in ND B-ALL patients who relapsed compared with that in ND patients in CCR

Firstly, we determined *DNMT3A* expression in 102 newly diagnosed (ND) B-ALL patients and 11 patients in continuous CR (control) by relative quantitative PCR. As a result, *DNMT3A* expression in ND patients with B-ALL, ranged from 0.0006594 to 1.713 with a median of 0.4363, was significantly higher than that in control patients (range: 0.08055 to 0.1865, median: 0.1147; *P* = 0.0004, Fig. 2a). Interestingly, *DNMT3A* expression in ND B-ALL patients who got relapse was significantly decreased compared with that in patients who were in CCR at the last follow-up (*P* = 0.0111, Fig. 2b).

### Low expression of *DNMT3A* indicated poor prognosis in ND B-ALL patients

**Fig. 3:**
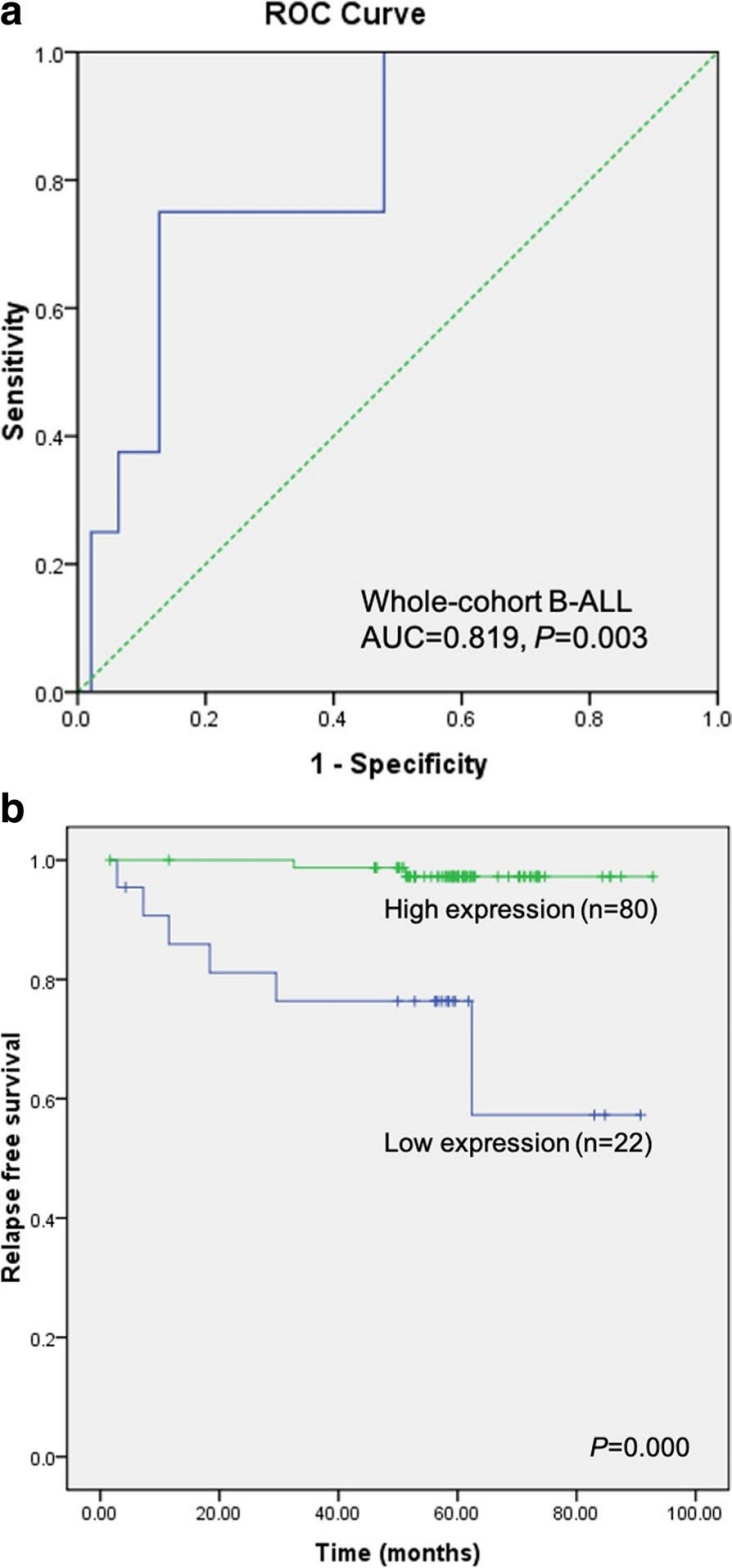
Prognostic significance of *DNMT3A* expression in 102 children with B-ALL. **a** ROC curve analysis of *DNMT3A* expression with relapse as an event. **b** The patients in *DNMT3A*^low^ group had poorer RFS than those in *DNMT3A*.^high^ group (*P* < 0.001)

ROC curve analysis was performed to evaluate the prognostic value of *DNMT3A* expression. When regarded as a continuous value, DNMT3A expression had a good predictive significance for relapse of B-ALL in children, with an area under curve (AUC) of 0.819 (95% CI: 0.686–0.952; *P* = 0.003), Fig. 3A), which indicated that *DNMT3A* expression could be a potential prognostic biomarker for ND B-ALL patients.

According to the ROC curve, 0.197 was determined as the optimal cutoff value for *DNMT3A* expression level, with a sensitivity and specificity of 75% and 87.2% respectively. Using this cut-off, we divided 102 patients into two groups, 22 cases with low *DNMT3A* expression (≤ 0.197, *DNMT3A*^low^) and 80 cases with high *DNMT3A* expression (> 0.197, *DNMT3A*^high^). There was a significantly higher relapse rate in *DNMT3A*^low^ group (6 out of 22 vs 2 out of 88, Fisher’s exact test, *P* = 0.001). Moreover, poor RFS was also observed in the *DNMT3A*^low^ group (57.3% ± 17.9% vs. 97.2% ± 1.9%, *P* < 0.001) (Fig. 3b). Among other common clinical features, only clinical risk classification had a trend of correlation with RFS (*P* = 0.053, Table 2), indicating its important prognostic value.

**Table 2:** Multivariate analysis of prognostic factors for relapse-free survival in children with B-cell acute lymphoblastic leukemia

Variables	Relapse-free survival				
	**Univariate**^a^ ***P***	**Multivariate**^b^			
		**Hazard ratio (HR)**	**95% CI for HR**		***P***
			**Lower**	**Upper**	
*DNMT3A* expression: Low (*n* = 21) vs. High (*n* = 75)	< 0.001	36.824	3.746	362.033	0.002
Age(years): < 1 or ≥ 10 (*n* = 7) vs. 1 ~ 10 (*n* = 89)	0.375	0.000	0.000		0.988
WBC counts at diagnosis (× 10^9^/L): ≥ 50 (*n* = 19) vs. < 50(*n* = 77)	0.16	1.042	0.21	5.178	0.96
MRD at day33: ≥ 10^–3^ (*n* = 11) vs. < 10^–3^ (*n* = 85)	0.172	0.969	0.098	9.525	0.978
Clinical risk:	0.053				
Intermediate-risk (*n* = 71) vs. Low-risk (*n* = 19)		1.757	0.198	15.624	0.613
High-risk (6) vs. Low-risk (*n* = 19)		64.978	1.837	2298.197	0.022

*HR* Indicates Hazard ratio, *WBC* White blood count, *MRD* Minimal residual disease

^a^Univariate analysis was performed by Kaplan–Meier Log-rank test

^b^All factors in the univariate analysis were selected in Cox regression of the multivariate analysis

In multivariate analysis for prognosis (Table 2), *DNMT3A* expression, Age, WBC at diagnosis, MRD at day 33 and clinical risk classification were used as covariates in a cohort of 96 cases due to no MRD result about 6 patients. Adjusted for all these factors, *DNMT3A* expression remained an independent prognostic factor for RFS of patients with B-ALL (HR = 36.824, 95% CI: 3.746 ~ 362.033, *P* = 0.002, Table 2). In addition, high risk was also an independent prognostic factor for RFS (*P* = 0.022, Table 2). These findings indicated that low expression of *DNMT3A* in leukemic cells at diagnosis could be a useful indicator for disease relapse in childhood B-ALL.

### Comparison of clinical features between *DNMT3A*^low^ and *DNMT3A*^high^ patients

In the next step, we analyzed the correlation of *DNMT3A* expression with common clinical characteristics such as age, gender, white blood cell (WBC) count at diagnosis and fusion genes. However, no correlation was found between *DNMT3A* expression and above clinical characteristics (Table 3).

**Table 3:** Correlation of *DNMT3A* expression with clinical characteristics in BCP-ALL

	***DNMT3A***^**low**^**, n (%)**	***DNMT3A***^**high**^**, n (%)**	***P***
**Age**			
< 1 or ≥ 10	1(4.5)	6 (7.5)	1.000
1 ~ 10	21(95.5)	74 (92.5)	
**Gender**			
Male	16 (72.7)	48 (60)	0.327
Female	6 (27.3)	32 (40)	
WBC(*10^**9**^**/L)**			
< 50	15 (68.2)	68 (85)	0.118
≥ 50	7 (31.8)	12 (15)	
**Fusion gene**			
Negative	19 (86.4)	49 (61.25)	0.264
*ETV6-RUNX1*	3 (13.6)	25 (31.25)	
*TCF3-PBX1*	0 (0)	3 (3.75)	
*BCR-ABL1*	0 (0)	2 (2.5)	
*FUS-ERG*	0 (0)	1 (1.25)	
**MRD at d33**			
< 10^–3^	18(85.7)	67 (89.3)	0.701
≥ 10^–3^	3(14.3)	8(10.7)	
**MRD at d78**			
< 10^–4^	21 (100)	72 (96)	1.000
≥ 10^–4^	0(0)	3(4)	
**Risk classification**			
Standard risk	6 (27.3)	16 (20)	0.708
Intermediate risk	15 (68.2)	58 (72.5)	
High risk	1 (4.5)	6 (7.5)	

We further analyzed the association of *DNMT3A* expression with MRD at d33, MRD at d78 and risk classification respectively, but no significant correlation between them was found (Fisher’s exact test, *P* > 0.05, Table 3).

### Knock-out of *DNMT3A* enhanced resistance of Reh and 697 cell lines to DNR

**Fig. 4:**
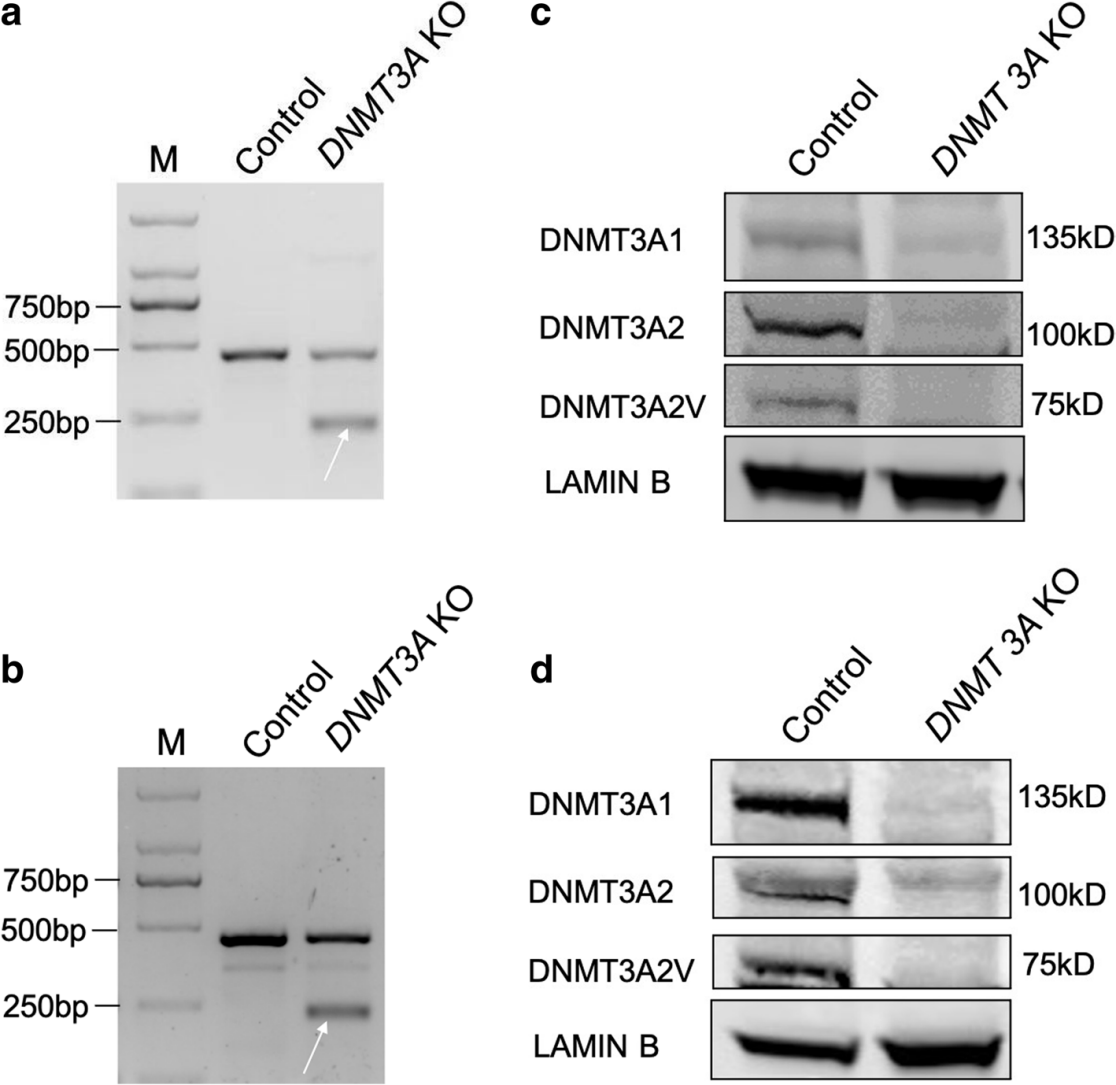
LentiCRISPR/Cas9 mediated editing of *DNMT3A* gene in Reh and 697 cell lines. **a** and **b** T7e1 assay analysis of specific sgRNA-mediated indels at DNMT3A locus in Reh and 697 cell lines separately. The lower migrating bands marking by a white arrow represent the disrupted gene alleles. **c** and **d** Expression of three DNMT3A protein variants was significantly reduced in Reh and 697 cell lines infected by *DNMT3A*-sgRNA lentivirus separately

To confirm the correlation of low expression of *DNMT3A* with poor prognosis of children with B-ALL, firstly, we disrupted *DNMT3A* in Reh and 697 cell lines separately. T7 endonuclease I (T7EN1) assay showed high efficiency of the sgRNA to direct Cas9-mediated ablation of *DNMT3A* (Fig. 4a and b). Furthermore, as expected, Western blotting indicated that *DNMT3A* expression was remarkably reduced after infection with *DNMT3A*-sgRNA lentivirus (Fig. 4c and d).

**Fig. 5:**
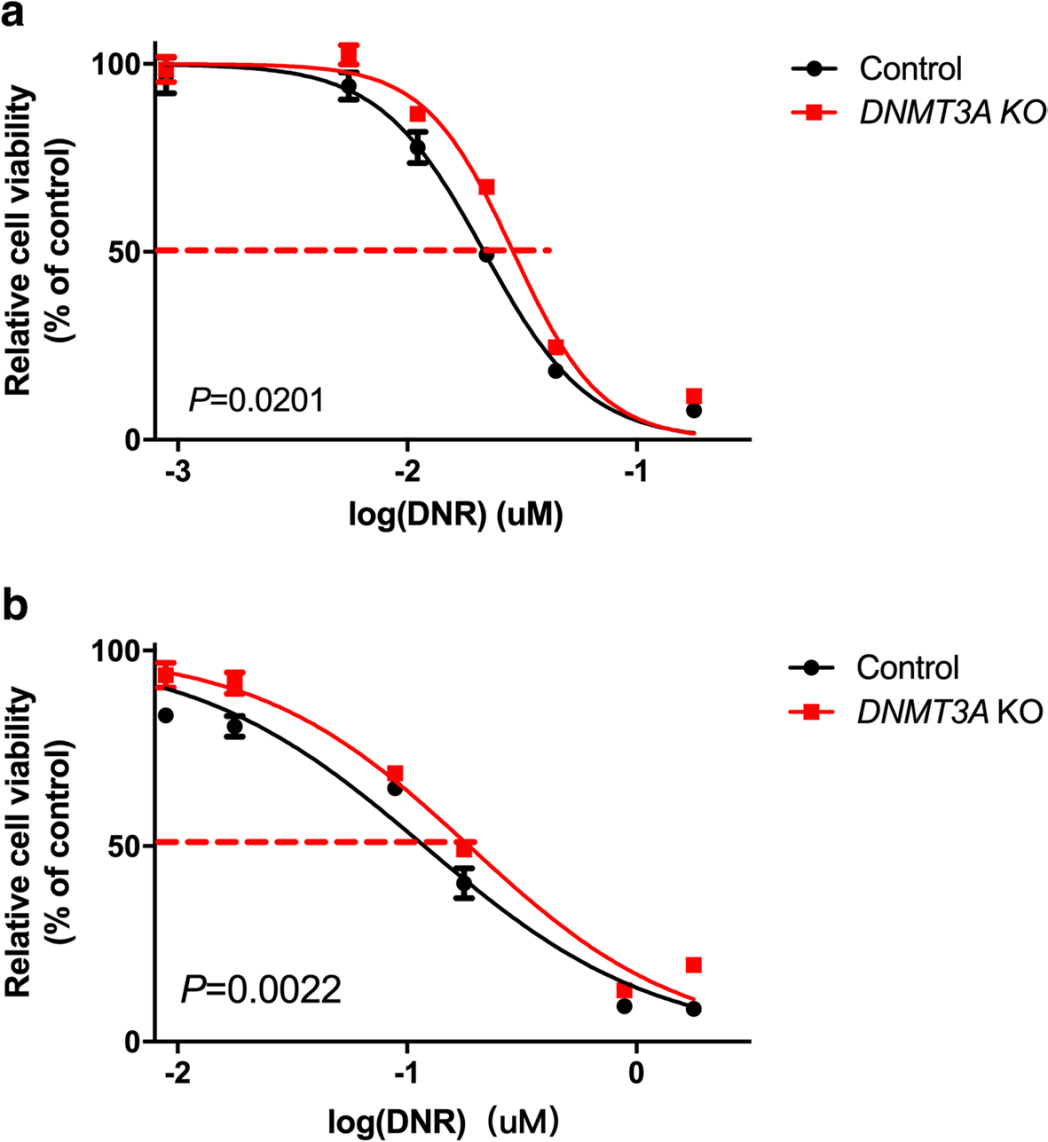
Knock-out of *DNMT3A* gene increased resistance of Reh and 697 cell lines to DNR. IC50 of DNR significantly increased in *DNMT3A*-knockout cells (Independent-samples T test, *P* = 0.0201 in Reh cell line (**a**) and *P* = 0.0022 in 697 cell line (**b**)). The standard errors of the means are shown (*n* = 3 experiments for each drug concentration)

DNR is one of the main chemotherapeutic drugs in induction therapy of B-ALL. We next tested whether knock-out of *DNMT3A* gene could cause Reh and 697 cell lines to be tolerant to DNR by CCK8 assay. These cells were treated by different concentrations of DNR for 24 h. It was shown that IC50 was significantly increased in the *DNMT3A*-knockout cells, indicating decreased cell viability (Fig. 5a, Control vs. *DNMT3A* KO, 0.02159 vs. 0.02892 μM, *P* = 0.0201; Fig. 5b, Control vs. *DNMT3A* KO, 0.1190 vs. 0.1865 μM, *P* = 0.0022). These results demonstrated that sgRNA mediated Cas9 knock-out of *DNMT3A* can causes Reh and 697 cell lines to be resistant to DNR, implying that *DNMT3A* expression plays an important role in the sensitivity of B-ALL leukemic cells to chemotherapeutic drugs such as DNR.

### Discussion

In recent two decades, *DNMT3A* mutations have been found in approximately 20% of adult AML patients, 9% of adult T-ALL and 0 ~ 1.4% of childhood AML, and the hotspots of mutations are mainly located in exon 23 which encodes the catalytic methyltransferase domain [5–11]. *DNMT3A* mutations are associated with poor prognosis and used for risk stratification in AML [5–10], and is associated with increased age and adverse outcome in adult T-ALL [11]. However, few studies focused on the role of *DNMT3A* in B-ALL. Our previous study has shown that *DNMT3A* mutations can be found in exon 23 and its adjacent intron regions in a few of children with B-ALL (5/182, 2.7%), and may have adverse impact on prognosis [12].

As there are only a few B-ALL patients with *DNMT3A* mutations, we sought to determine the prognostic significance of *DNMT3A* expression in B-ALL. As expected, low expression was associated with relapse in 102 patients with B-ALL. Furthermore, knock-out of *DNMT3A* increased IC50 of DNR in Reh and 697 cell lines, indicating the relationship of low expression of *DNMT3A* and chemoresistance.

It was reported that Dnmt3a loss in HSCs leads to hypomethylation of genes with a causal role in cancer, such as *Runx1* and *Gata3*. Runx1 promotes murine erythroid progenitor proliferation and inhibits differentiation by preventing Pu.1 downregulation [23]. Gata3 targets Runx1 in the embryonic hematopoietic stem cell niche [24]. Thus, previous studies and ours’ suggest that deletion or low-expression of *DNMT3A* result in differentiation inhibition of HSCs and allow HSCs to be propagated indefinitely in vivo [25–28], which may play an important role in leukemogenesis and resistance to chemotherapy. This may provide us with an alternative target of therapy for childhood B-ALL.

It has been reported that *DNMT3A* expression is directly transactivated by transcription factor WT1 in Wilms’ tumor cells [29] and is negatively regulated by p53 at the transcriptional level in lung cancer [30]. In fact, overexpression of the WT1 transcript was demonstrated in children with B-ALL at diagnosis [31], which may contribute to increased expression of *DNMT3A* in leukemic cells. However, increased expression of *TP53* by 2 to 20-fold higher in pediatric primary B-ALL than in healthy controls [32] would inhibit *DNMT3A* expression. Thus, the regulation mechanism of *DNMT3A* expression is quite complicated in different types of cancer cells and needs to be clarified especially in childhood B-ALL.

There are some limitations or weakness in this study. Firstly, as this is a retrospective study and only patients with > 70% of bone marrow infiltration were selected, the findings were based on a small number of cases. A prospective study with large sample size and long-term follow-up are necessary to confirm the prognostic significance of DNMT3A. Secondly, the influence of treatment compliance could not be assessed. More attention should be paid to this point in the prospective study. Thirdly, we only focused on DNMT3A expression, ignoring its DNA methyltransferase activity, the role of which in chemoresistance of B-ALL cells should be clarified in future study.

## Conclusions

In summary, we associated low expression of *DNMT3A* with poor prognosis in Chinese pediatric patients with B-ALL. Furthermore, the knockout of *DNMT3A* conferred less sensitivity to daunorubicin in leukemic cell lines. Successful disruption of *DNMT3A* in Reh and 697 cell lines may facilitate the studies on mechanism of relapse and chemotherapeutic resistance for childhood B-ALL. Future prospective studies with large sample size, long-term follow-up, more leukemia cell lines and more mechanism research were recommended to confirm the DNMT3A role in childhood with B-ALL.

## Supplementary Information

**Additional file 1: Table S1.** Comparison of clinical characteristics in children with B-cell acute lymphoblastic leukemia include or excluded in this study.

## Data Availability

All data generated and analyzed during this study are included in this manuscript and original data as supplemental part.

## Abbreviations

B-ALL: B cell acute lymphoblastic leukemia
RFS: Relapse-free survival
DNR: Daunorubicin
CCR: Continuous complete remission
DNMT3A: DNA methyltransferase 3A
AML: Acute myeloid leukemia
BM: Bone marrow
CCLG-ALL 2008: Chinese Children’s Leukemia Group ALL 2008 Protocol
CR: Continuous complete remission
Ig/TCR: *Immunoglobulin* And *T cell receptor* gene rearrangements
MRD: Minimal residual disease
RQ-PCR: Real-time quantitative polymerase chain reaction
ROC: Receiver Operating Characteristic
RFS: Relapse free survival
WBC: White blood cell
T7EN1: T7 endonuclease I
