# Correction to: DNMT3A low-expression is correlated to poor prognosis in childhood B-ALL and confers resistance to daunorubicin on leukemic cells

**DOI:** 10.1186/s12885-023-10862-x

**Published:** 2023-04-21

**Authors:** Weijing Li, Shuguang Liu, Chanjuan Wang, Lei Cui, Xiaoxi Zhao, Wei Liu, Ruidong Zhang, Zhigang Li

**Affiliations:** 1 Laboratory of Hematologic Diseases, Beijing Children’s Hospital, Beijing Pediatric Research Institute, Capital Medical University, National Center for Children’s Health, Beijing, China; 2 Beijing Key Laboratory of Pediatric Hematology-Oncology, Beijing, China; 3 National Key Discipline of Pediatrics, Capital Medical University, Beijing, China; 4 Key Laboratory of Major Diseases in Children, Ministry of Education, Beijing, China; 5 Hematology Oncology Center, Beijing Children’s Hospital, Capital Medical University, National Center for Children’s Health, Beijing, China; 6 Department of Hematology Oncology, Children’s Hospital Affiliated to Zhengzhou University, Zhengzhou, China

**Correction: BMC Cancer 23, 255 (2023)**.

10.1186/s12885-023-10724-6

Following publication of the original article [1], the authors identified an error in the given name of author Shuguang Liu.

The incorrect author name is: Shugang Liu.

The correct author name is: Shuguang Liu.

The author group has been updated above and the original article [1] has been corrected.
