# Supplementary material for: DNMT3A low-expression is correlated to poor prognosis in childhood B-ALL and confers resistance to daunorubicin on leukemic cells
Source: BMC Cancer. 2023 Mar 18;23:255. doi: 10.1186/s12885-023-10724-6 (PMC10024838; doi:10.1186/s12885-023-10724-6)
Supplement: Supplementary file 1 — Additional file 1: Table S1. Comparison of clinical characteristics in children with B-cell acute lymphoblastic leukemia include or excluded in this study. [file 12885_2023_10724_MOESM1_ESM.doc]

Table S1 Comparison of clinical characteristics in children with B-cell acute lymphoblastic leukemia include or excluded in this study.

| Variables | Total  (226) | Include  (n=102) | Excluded  (n=124) | *P*-values |
| --- | --- | --- | --- | --- |
| Age(years) at diagnosis, median(range) | 4.0 (0.7-15.9) | 3.8(1-13.3) | 4.2(0.7-15.9) | 0.390^a^ |
| Gender, male, n (%) | 134(59.3) | 64(62.7) | 70(56.5) | 0.235^b^ |
| WBC count (*10^9^/L), median(range) | 12.03(1.42-680.39) | 13.06(1.5-680.39) | 11.17(1.42-608.97) | 0.947 ^a^ |
| Fusion gene |  |  |  |  |
| *ETV6-RUNX1* | 55(24.3) | 28(27.5) | 27(21.2) | 0.202 ^b^ |
| *TCF3-PBX1* | 11(4.9) | 3(2.9) | 8(6.5) | 0.353 ^b^ |
| *BCR-ABL1* | 7(3.1) | 2(2.0) | 5(4) | 0.462 ^b^ |
| *FUS-ERG* | 2(0.9) | 1(1.0) | 1(0.8) | 1.000 ^b^ |
| Relapse (%) ^c^ | 18(92) | 8(92.2) | 10(91.9) | 1.000 ^b^ |

^a^ Mann-Whitney U test for continuous variable subgroups for age and WBC.

^b^ Fisher’s exact test for categorical subgroups.
